# Performance of ChatGPT 4.0 on Japan's National Physical Therapist Examination: A Comprehensive Analysis of Text and Visual Question Handling

**DOI:** 10.7759/cureus.67347

**Published:** 2024-08-20

**Authors:** Shogo Sawamura, Kengo Kohiyama, Takahiro Takenaka, Tatsuya Sera, Tadatoshi Inoue, Takashi Nagai

**Affiliations:** 1 Department of Rehabilitation, Heisei College of Health Sciences, Gifu, JPN

**Keywords:** images and tables, text-only questions, national physical therapist exam, chatgpt 4.0, large-scale language model

## Abstract

Introduction: ChatGPT 4.0, a large-scale language model (LLM) developed by OpenAI, has demonstrated the capability to pass Japan's national medical examination and other medical assessments. However, the impact of imaging-based questions and different question types on its performance has not been thoroughly examined. This study evaluated ChatGPT 4.0's performance on Japan's national examination for physical therapists, particularly its ability to handle complex questions involving images and tables. The study also assessed the model's potential in the field of rehabilitation and its performance with Japanese language inputs.

Methods: The evaluation utilized 1,000 questions from the 54^th^ to 58^th^ national exams for physical therapists in Japan, comprising 160 general questions and 40 practical questions per exam. All questions were input in Japanese and included additional information such as images or tables. The answers generated by ChatGPT were then compared with the official correct answers.

Analysis: ChatGPT's performance was evaluated based on accuracy rates using various criteria: general and practical questions were analyzed with Fisher's exact test, A-type (single correct answer) and X2-type (two correct answers) questions, text-only questions versus questions with images and tables, and different question lengths using Student's t-test.

Results: ChatGPT 4.0 met the passing criteria with an overall accuracy of 73.4%. The accuracy rates for general and practical questions were 80.1% and 46.6%, respectively. No significant difference was found between the accuracy rates for A-type (74.3%) and X2-type (67.4%) questions. However, a significant difference was observed between the accuracy rates for text-only questions (80.5%) and questions with images and tables (35.4%).

Discussion: The results indicate that ChatGPT 4.0 satisfies the passing criteria for the national exam and demonstrates adequate knowledge and application skills. However, its performance on practical questions and those with images and tables is lower, indicating areas for improvement. The effective handling of Japanese inputs suggests its potential use in non-English-speaking regions.

Conclusion: ChatGPT 4.0 can pass the national examination for physical therapists, particularly with text-based questions. However, improvements are needed for specialized practical questions and those involving images and tables. The model shows promise for supporting clinical rehabilitation and medical education in Japanese-speaking contexts, though further enhancements are required for a comprehensive application.

## Introduction

ChatGPT is a large-scale language model (LLM) developed by OpenAI, and it was released for public use in November 2022. It utilizes an extensive corpus of text data to predict the next word and generates text accordingly [1]. ChatGPT can perform various tasks beyond text generation [2], and it is being applied in multiple fields. In the medical domain, it has the potential to be a robust tool to address healthcare disparities in low-resource countries, with applications in patient screening, diagnostic support, treatment assistance, health indicator tracking, and community education [3]. Furthermore, ChatGPT can serve as a decision-support tool for diagnosis and treatment [4,5] and for generating medical documents, thereby optimizing limited medical resources [5,6]. In the field of rehabilitation, there have been attempts to apply it in clinical reasoning [7], rehabilitation program planning [8,9], and patient management [10,11].

LLMs are expected to have substantial potential in medical education. For instance, conversational agents powered by LLMs can provide personalized learning experiences to enhance the critical thinking and problem-solving skills of students [12]. ChatGPT has been utilized in medical education [13], and recent efforts have focused on generating multiple-choice questions [14,15]. Such applications can significantly improve the quality and efficiency of medical education [16,17].

However, LLMs pose risks of generating misinformation including hallucinations [18,19]. ChatGPT must exhibit high performance to be effectively utilized in the medical field [20]; it should at least be able to pass relevant national examinations. Prior studies have reported that ChatGPT achieves commendable results in national medical exams [16,21-25]. Nevertheless, there are only a few cases where it has successfully answered complex questions, such as those including images and tables [26].

This study aims to evaluate the extent to which ChatGPT can handle complex exams, including questions with images and tables, with a focus on its application in the rehabilitation field. Specifically, the study aims to ascertain whether ChatGPT can achieve sufficient performance to pass the complete national examination for physical therapists, including questions with images and tables, and to identify the factors that affect its performance. ChatGPT 4.0 provides improved support for non-English languages, including Japanese [27]. Thus, this study also assesses its performance using inputs and outputs entirely in Japanese considering its application in Japan, where clinical practice and exams are predominantly conducted in Japanese.

## Materials and methods

Overview of the national examination for physical therapists in Japan

**Figure 1:**
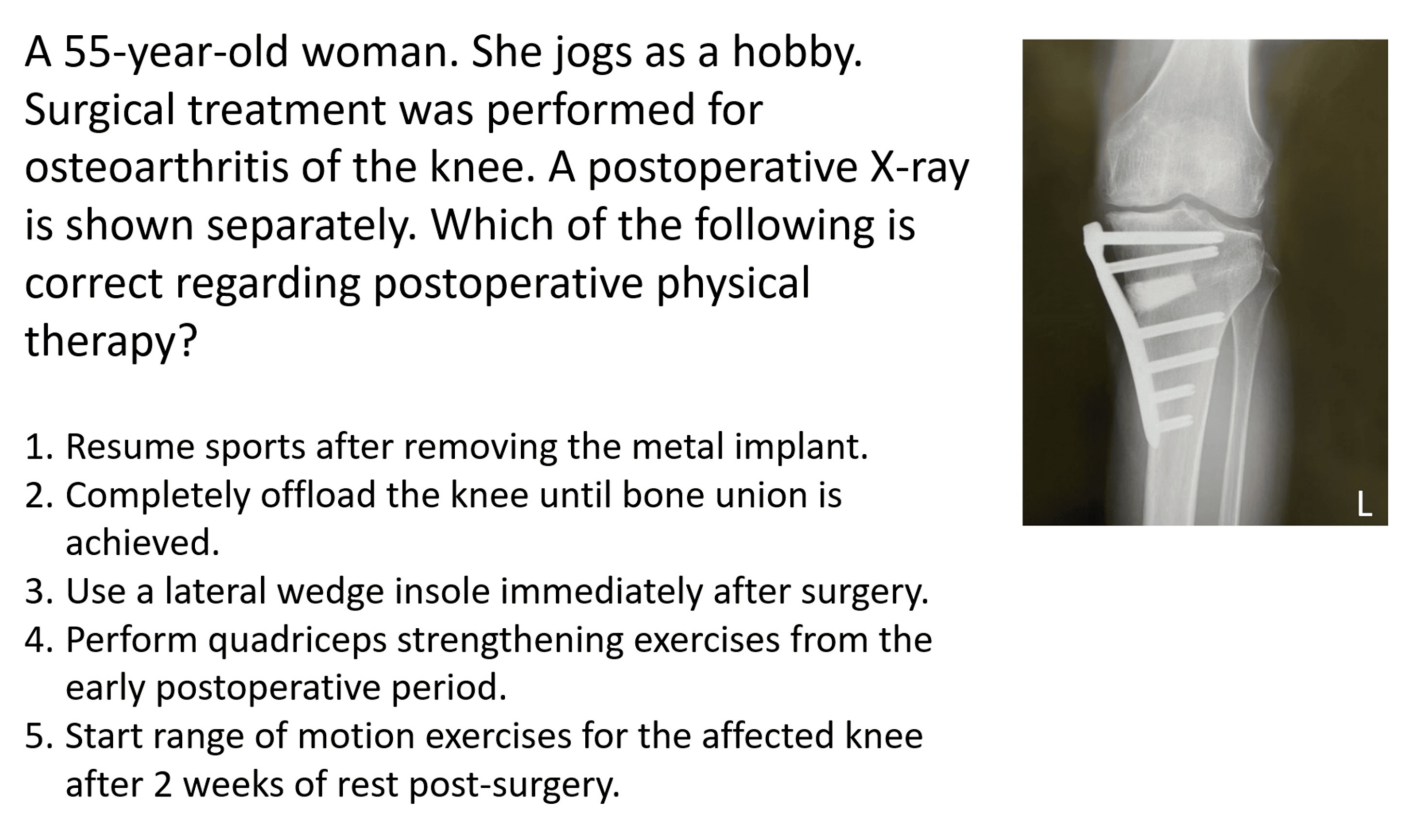
Practical question from the National Examination for Physical Therapists Translated from Japanese to English from the 58th National Examination for Physical Therapists

The national examination for physical therapists in Japan comprises multiple-choice questions, including 160 general questions and 40 practical questions, worth 1 and 3 points each, respectively. General questions cover subjects such as anatomy, physiology, kinesiology, pathology, clinical psychology, rehabilitation medicine, clinical medicine, and physical therapy. Practical questions focus specifically on physical therapy and require specialized knowledge. For example, some questions provide a brief case summary and ask the examinee to select the appropriate physical therapy intervention (see Figure 1). Other typical questions are listed in the appendix. Both general and practical questions may include images or tables. The exam features A-type questions, where an examinee selects one correct answer from five choices, and X2-type questions, where two correct answers must be selected from five choices. The passing criteria vary by exam session but generally require an overall score of approximately 60% or higher and a certain percentage of correct answers in practical questions.

Input to ChatGPT

This study utilized 1,000 questions from the 54th to 58th National Examinations for Physical Therapists in Japan. Twenty questions excluded by the Ministry of Health, Labour, and Welfare due to inappropriate content were not included in the analysis. The questions were input into ChatGPT 4.0 as text data in Japanese (zero-shot prompting). Additional information, such as images or tables, was input along with the text data. The correctness of the answers obtained using ChatGPT was verified against the official correct answers published by the Ministry of Health, Labour, and Welfare. Data were collected from January 1-4, 2024.

Analysis

The performance of ChatGPT 4.0 was compared with the passing criteria published by the Ministry of Health, Labour, and Welfare to determine if it met the requirements for passing the national examination for physical therapists. Detailed analyses were conducted as follows:

General vs. practical questions: The accuracy rates for general and practical questions were compared using Fisher's exact test to examine the impact of different specialization levels on ChatGPT's performance.

Question types: X2-type questions are generally considered more challenging due to the lower probability of correct guesses compared to A-type questions. The impact of question format difficulty on ChatGPT's performance was examined by comparing the accuracy rates for these questions using Fisher's exact test.

Ability to interpret images and tables: As ChatGPT may find it difficult to interpret medical images, the impact of the presence of images on its performance was examined by comparing the accuracy rates for text-only questions and questions with images and tables using Fisher's exact test.

Number of characters in questions: The amount of information provided in a question might affect ChatGPT's accuracy. The impact of question length on its performance was examined by comparing the number of characters in correctly and incorrectly answered questions using Student's t-test.

## Results

**Figure 2:**
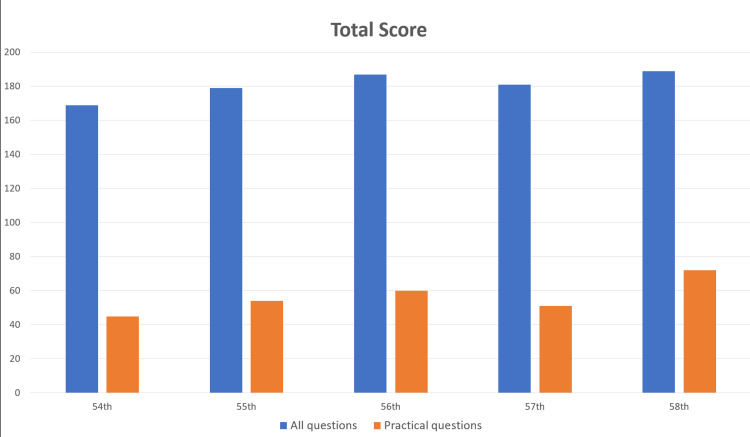
Scores for the 54th-58th National Examinations for Physical Therapists

**Figure 3:**
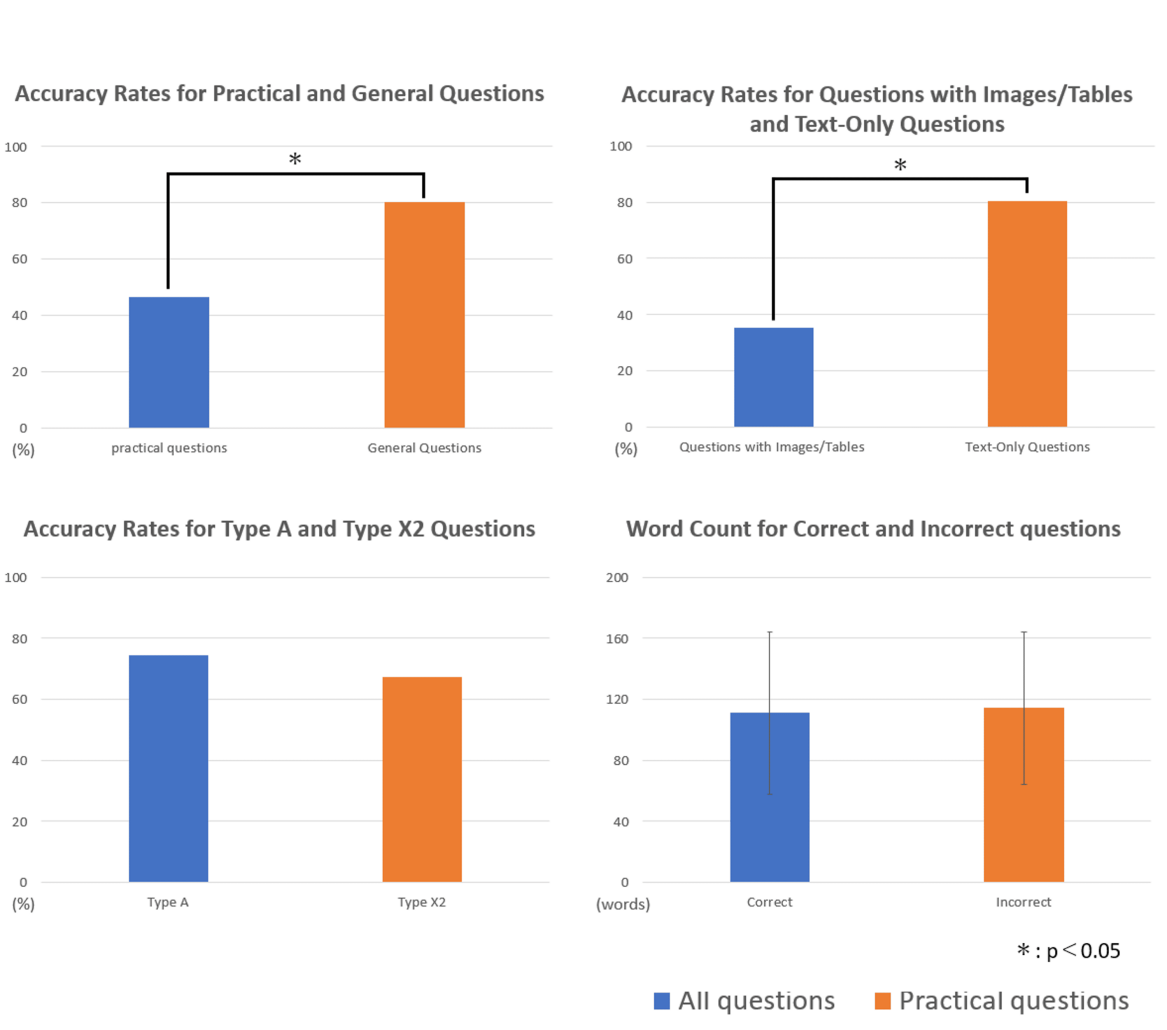
Detailed analysis of ChatGPT's performance on the National Examination for Physical Therapists

The overall results are summarized in Tables 1-2 and Figure 2. ChatGPT 4.0 met the passing criteria for all exams, achieving an overall accuracy rate of 73.4% (717/980). Detailed analysis results are shown in Figure 3.

**Table 1:** Scores obtained by ChatGPT 4.0 for the 54th to 58th National Examinations for Physical Therapists

		54^th^	55^th^	56^th^	57^th^	58^th^	Total
All Questions	Number of Questions	192	197	197	196	198	980
	Correct	139	143	147	147	141	717
	Accuracy Rate	0.72395833	0.72588832	0.74619289	0.75	0.71212121	0.733825
Practical Questions	Number of Questions	39	40	39	38	40	196
	Correct	15	18	20	17	24	94
	Accuracy Rate	0.38461538	0.45	0.51282051	0.44736842	0.6	0.465909
General Questions	Number of Questions	153	157	158	158	158	784
	Correct	124	125	127	130	117	623
	Accuracy Rate	0.81045752	0.79617834	0.80379747	0.82278481	0.74050633	0.800709
Questions Including Images and Tables	Number of Questions	38	30	27	31	25	151
	Correct	13	10	8	12	12	55
	Accuracy Rate	0.34210526	0.33333333	0.2962963	0.38709677	0.48	0.353791
Text-Only Questions	Number of Questions	154	167	170	165	173	829
	Correct	126	133	139	135	129	662
	Accuracy Rate	0.81818182	0.79640719	0.81764706	0.81818182	0.74566474	0.804714
Type A	Number of Questions	167	175	172	165	168	847
	Correct	124	127	132	125	118	626
	Accuracy Rate	0.74251497	0.72571429	0.76744186	0.75757576	0.70238095	0.743119
Type X2	Number of Questions	25	22	25	31	30	133
	Correct	15	16	15	22	23	91
	Accuracy Rate	0.6	0.72727273	0.6	0.70967742	0.76666667	0.673729

**Table 2:** Scores and passing criteria by ChatGPT 4.0 for the 54th to 58th National Examinations for Physical Therapists

		54^th^	55^th^	56^th^	57^th^	58^th^
Total Score	Practical Questions	45	54	60	51	72
	General Questions	124	125	127	130	117
	Total	169	179	187	181	189
Passing Criteria	Practical Questions	41	43	41	40	43
	Total Score	164	167	135	164	167

The accuracy rates for general and practical questions were 80.1% (623/784) and 46.6% (94/196), respectively, showing a significant difference (p < 0.05). The accuracy rates for A-type and X2-type questions were 74.3% (626/847) and 67.4% (91/133), respectively, with no significant difference. The accuracy rates for text-only questions and questions with images and tables were 80.5% (662/829) and 35.4% (55/151), respectively, showing a significant difference. There was no significant difference in the number of characters between correctly answered questions (111.0 ± 53.4 words) and incorrectly answered questions (114.3 ± 50.0 words).

## Discussion

The results indicate that ChatGPT 4.0 meets the passing criteria for the 54th-58th National Examinations for Physical Therapists. This suggests that ChatGPT 4.0 exhibits the performance required to pass the national examination. The exam consists of multiple-choice questions that assess not only memory but also understanding, application, analysis, and evaluation skills [28,29]. Thus, ChatGPT 4.0 may possess the minimum required knowledge, understanding, and application skills necessary for a physical therapist. Previous studies have reported that ChatGPT passes Japan's national medical examination [20] and other medical exams, such as the US medical licensing exams, with commendable results [16,21-24]. However, these studies excluded questions with images and tables and did not test ChatGPT on a complete exam. The results of this study are notable because ChatGPT successfully passed the entire exam, including questions with images and tables.

The accuracy rate for practical questions is significantly lower than that for general questions. Practical questions require more specialized knowledge in physical therapy. Although ChatGPT generates answers based on extensive learning data, it may lack sufficient information in highly specialized fields, resulting in lower accuracy rates for practical questions. There is no significant difference between the accuracy rates for A-type and X2-type questions, indicating that ChatGPT's performance is not strongly influenced by question format difficulty but rather by the essential knowledge required.

The accuracy rate for questions with images and tables is significantly lower than that for text-only questions. This suggests that ChatGPT struggles to interpret images, which is consistent with previous studies [26,30]. There is no significant difference in the number of characters between correctly and incorrectly answered questions, aligning with findings that ChatGPT's accuracy for multiple-choice questions is not related to the length of the question text [21].

The evaluation of ChatGPT's performance with Japanese inputs yielded good results, despite the model being developed in the US with a predominantly English-language corpus [27]. This suggests that ChatGPT can perform well with non-English inputs, highlighting its potential benefits for non-English-speaking medical professionals.

This study has three limitations. First, ChatGPT's performance may vary with different versions, so it is unclear if the results observed in this study apply to the current version. Second, the national examination for physical therapists in Japan consists solely of multiple-choice questions and does not include other formats, such as descriptive or argumentative questions. Therefore, this exam may not fully assess ChatGPT's overall performance. Third, the study does not provide a detailed analysis of the questions answered incorrectly. If incorrect answers are due to gaps in ChatGPT's knowledge, the patterns of incorrect responses might differ across various fields.

In the future, it will be important to evaluate the performance of new versions of the model and assess its capabilities on written and argumentative examinations. Additionally, analyzing incorrectly answered questions will help identify areas where ChatGPT may exhibit poor performance.

## Conclusions

ChatGPT 4.0 meets the performance requirements to pass the national examination for physical therapists, including questions with images and tables. Its performance is not significantly affected by question format or text length but is generally lower for more specialized practical questions and questions with images and tables. While ChatGPT 4.0 performs effectively with text-based questions, highlighting its potential as a tool for clinical rehabilitation and medical education, it is important to note that it does not answer all questions correctly. Additionally, ChatGPT 4.0 demonstrates strong performance with Japanese inputs, suggesting its usefulness for non-English-speaking medical professionals.

## Figures and Tables

**Figure 4:**
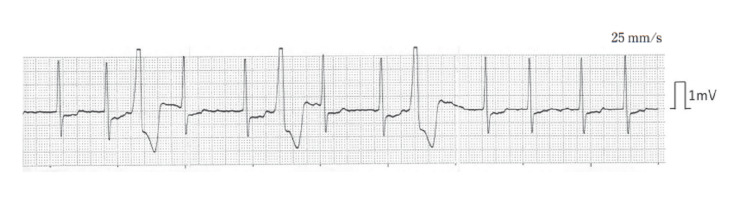
The ECG waveform of Q1 Image taken from 54th-58th National Examinations for Physical Therapists

**Figure 5:**
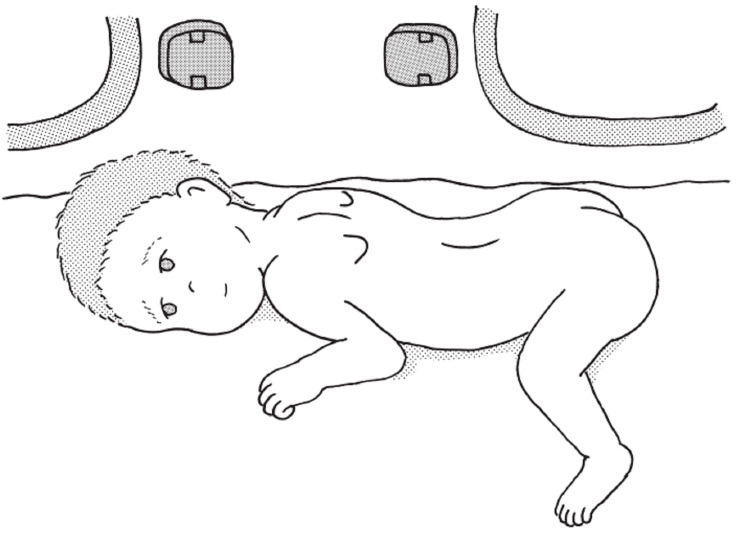
Image of the supine position of a low-birth-weight baby in Q3 Image taken from 54th-58th National Examinations for Physical Therapists

**Figure 6:**
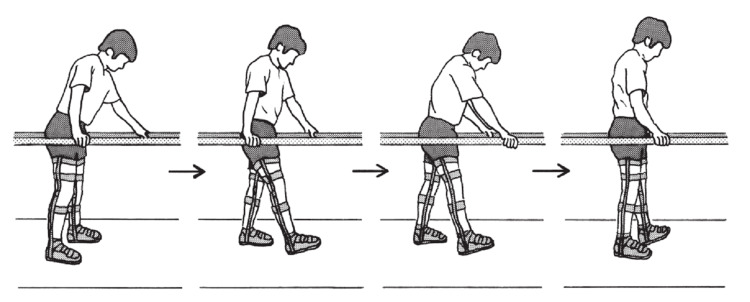
Image of a 28-year-old man with complete spinal cord injury using bilateral long leg braces and practicing walking with parallel bars Image taken from 54th-58th National Examinations for Physical Therapists

## Appendices

**Table 3:** Typical questions in 54th-58th National Examinations for Physical Therapists Translated from Japanese to English from 54th-58th National Examinations for Physical Therapists

Question Type	Question	Answer
Practical Questions	Q1: The ECG waveform is shown separately (Fig. 4). Which of the following is the correct characteristic? 1. Sinus rhythm. 2. Sustained tachycardia. 3. ST-segment elevation is observed. 4. Ventricular extrasystole is observed. 5. The patient has a third-degree atrioventricular block.	4
Practical Questions	Q2: A 68-year-old man was diagnosed with Parkinson's disease 5 years ago. Currently, he experiences resting tremors in both hands and moderate muscle stiffness in both upper and lower limbs. He reports, "Lately, I have difficulty getting to my feet when I walk, and I increasingly lose my balance, almost falling. Although I am independent in my daily activities, I avoid going outside because I am concerned about falling while walking outdoors." Based on this description, which stage of the Hoehn & Yahr severity classification applies to this patient? 1. I 2. II 3. III 4. IV 5. V	3
Practical Questions	Q3: A low birth weight baby, born at 30 weeks gestational age, has been admitted to the NICU. The supine position is shown in the figure 5. Which two positions are appropriate for positioning this infant? Select the two correct options. 1. Head extended 2. Trunk in extension 3. Shoulder girdle protruding forward 4. Shoulder in abduction 5. Hip joint in mid-rotation	3, 5
Practical Questions	Q4: A 28-year-old male with a complete spinal cord injury uses bilateral long leg braces and practices walking in parallel bars. His gait pattern is shown in the figure 6. What is the residual level of function? 1. Th1 2. Th6 3. Th12 4. L4 5. S1	3
Practical Questions	Q4: A 75-year-old male with a right lower limb amputation due to diabetes mellitus complains of instability in his right knee during prosthetic walking practice, which could lead to a knee buckle. What are the possible causes? Select the two correct options. 1. Excessive initial flexion angle. 2. Insufficient initial adduction angle. 3. Limited flexion range of motion of the right hip joint 4. Decreased right knee joint extensor strength 5. The socket is positioned too far posterior to the foot	1, 4
General Questions	Q5: Which of the following is true of muscle contraction? 1. during maximal contraction of skeletal muscle, the length of myocytes is shortened by about 10%. 2. Actin filaments are thicker than myosin filaments. 3. Muscle contraction is initiated by K+ release from the sarcoplasmic reticulum. 4. The enzyme that degrades ATP is present in action. 5. H-zone shortens during muscle contraction	5
General Questions	Q6: Which of the following branches is directly from the aortic arch? Choose two correct options. 1. Brachiocephalic artery 2. Right subclavian artery 3. Left subclavian artery 4. Right vertebral artery 5. Left vertebral artery	1, 3
General Questions	Q7: Which muscle contracts eccentrically when the body is slowly lowered from a suspension on an iron bar with the arms positioned at 90° shoulder flexion and 90° elbow flexion? 1. Supraspinatus 2. Vastus lateralis 3. Brachioradialis 4. Anterior deltoid 5. Pectoralis major clavicularis	2
General Questions	Q8: Which of the following is true of obsessive-compulsive disorder? 1. medication is not effective. 2. Exposure-response interference is used. 3. Compulsive behavior is due to the experience of being forced to do something. 4. Depression is a rare complication. 5. The patient is unaware of the irrationality of the compulsive behavior	2
General Questions	Q9: Which of the following constitutes the talus and articulation? Choose two correct options. 1. Calcaneus 2. Navicular bone 3. Cuboid bone 4. First metatarsal bone 5. Medial cuneiform	1
